# Ankle Foot Orthosis Intervention Improves the Ground Reaction Forces During Walking in Patients with Peripheral Artery Disease (Randomized Clinical Trial)

**DOI:** 10.3390/act15040187

**Published:** 2026-03-27

**Authors:** Zahra Salamifar, Farahnaz Fallahtafti, Kaeli Samson, Iraklis I. Pipinos, Jason M. Johanning, Sara A. Myers

**Affiliations:** 1 Department of Biomechanics, University of Nebraska at Omaha, Omaha, NE 68182, USA;; 2 Department of Biostatistics, University of Nebraska Medical Center, Omaha, NE 68198, USA;; 3 Department of Surgery, University of Nebraska Medical Center, Omaha, NE 68198, USA;; 4 Department of Surgery and VA Research Service, VA Nebraska-Western Iowa Health Care System, Omaha, NE 68105, USA;

**Keywords:** assistive device, gait, claudication, ischemia, walking performance, kinetics, peripheral vascular occlusive disease

## Abstract

This study investigated the impact of walking with ankle-foot-orthoses (AFOs) and without AFOs (non-AFO) on ground reaction forces (GRFs) in patients with peripheral artery disease (PAD). Additionally, this study examined the effect of AFO intervention vs. no AFO intervention on GRFs while walking with and without AFOs. Fifty patients with PAD were randomly assigned to either a three-month intervention (AFO) or a control (standard-of-care) group. After three months, subjects crossed over to the alternate group and were evaluated after three additional months. GRF data (anterior-posterior, medial-lateral, and vertical) were collected during walking with and without AFOs at baseline, three, and six months. Peak discrete GRF points, braking and propulsion impulses were compared across conditions, groups, and time points using linear mixed models. The peak brake and propulsion GRF were significantly reduced while walking with AFOs versus non-AFO (*p* < 0.01). Compared to non-AFO, walking with AFOs significantly reduced lateral GRF magnitude (*p* = 0.03) and significantly increased medial GRF (*p* = 0.02). The first and second maximum (*p* < 0.01) vertical GRF were significantly increased with AFOs versus non-AFOs. Walking with AFOs helped patients with PAD achieve greater peak propulsion and vertical GRFs compared to non-AFO, with GRF values trending toward those previously reported in healthy individuals.

## 1. Introduction

Peripheral artery disease (PAD) results from atherosclerotic blockages of the arteries in the lower extremities [1,2]. Intermittent claudication is the most common symptom of PAD, which occurs when the metabolic demand of the leg muscles during walking exceeds the oxygen supply, leading to ischemia and leg pain [3,4]. Claudication reduces mobility, physical activity, walking efficiency, and overall health outcomes. Furthermore, it increases oxygen uptake during pain-free walking compared to healthy individuals of a similar age [4–7]. Previous studies found that gait alterations occur even before the onset of claudication pain in both affected and unaffected legs [5,6,8–11]. Gait alterations in PAD include reduced ankle plantar flexor torques and powers, knee powers, hip powers, and step lengths [8,11]. Muscle atrophy, myopathy, and hip and ankle muscle weakness have also been reported [9,12–14]. The muscle strength deficit in the lower extremities, particularly in the calf muscles, invokes a progressive functional decline in patients who suffer from PAD compared to healthy older adults [8].

Ground reaction forces (GRFs) are important biomechanical parameters in gait analysis that provide insight into the mechanisms driving walking impairment in patients with PAD [4,15,16]. GRFs during walking reflect the neuromechanical function of the lower extremity joints and muscles. Properly analyzing GRFs during walking can provide information about the efficiency and safety of movement and the effectiveness of rehabilitation techniques [17,18]. GRFs are defined based on Newton’s third law of motion regarding action-reaction forces that affect the body while in contact with the ground. These forces are assessed in anterior-posterior, medial-lateral, and vertical directions [4,19]. Previous studies reported a detailed biomechanical gait analysis of patients with PAD and found several differences in their GRFs compared to controls of similar age without PAD [4,20]. Anterior-posterior and vertical GRFs were significantly reduced in patients with PAD compared to controls [4,8,9,19]. Patients with PAD also showed larger medial and smaller lateral GRFs compared to the control subjects [4]. Understanding these GRF disparities and how gait performance responds to interventions can promote understanding of treatment efficacy for individuals with PAD [21,22]. Another recent study showed that GRFs provided important information about classifying individuals as having or not having PAD, indicating GRFs’ importance as a gait indicator [7,23]. This research will enhance our understanding of the underlying mechanisms contributing to impaired locomotion in PAD and potentially improve clinical outcomes for these individuals.

Standard-of-care treatments for patients with PAD all address the blood flow limitations, but not the established muscle weakness [24]. There is a critical need for treatments that support the weakened and myopathic muscles during walking in patients with PAD [12,25]. One potential treatment approach to support muscle weakness in patients with PAD during walking is assistive devices, such as ankle-foot-orthoses (AFOs). AFOs have been shown to support weak calf muscles in neurological populations [26,27] by reducing the required ankle plantar flexor torque and power during walking [28]. Carbon composite AFOs absorb energy during weight acceptance and release stored energy during push-off, decreasing blood flow demand and muscle stress [29,30]. Previous studies found increased walking distances in patients with PAD while wearing AFOs [29,31]. Therefore, AFOs may be an effective intervention for improving functional performance in patients with PAD [7,25,29].

Given the limited understanding regarding the impact of AFO interventions on GRFs during walking in patients with PAD, this study has two primary objectives: (1) To assess how GRFs changed after a three-month AFO intervention, and (2) To assess the effect of walking with versus without AFOs in patients with PAD. These objectives will be addressed using a crossover study design. The subjects will be randomly assigned to two groups: those who received the AFO intervention first and those who received standard care. All the participants will be assessed during walking with and without AFOs before and after a three-month AFO intervention and standard care. We hypothesized that walking with AFOs would drive medial-lateral and vertical GRF profiles to improve, making them more comparable to control subjects. Additionally, we expect that a three-month AFO intervention would enhance push-off capabilities and reduce calf muscle demand by improving the propulsion GRF in the anterior-posterior direction generated during walking. Also, we anticipated that walking with AFOs would drive medial-lateral and vertical GRF profiles to improve, making them more comparable to those of healthy subjects.

## 2. Materials and Methods

This study involves a sub-analysis of a primary clinical trial. The sample size was determined using the initial claudication walking distance, which is not included in this study.

### Participants:

Fifty patients with PAD volunteered to participate in this study. However, 17 patients with PAD were excluded due to withdrawal and screen failure. The control first group started with 22 subjects, while the intervention first group started with 11 subjects. The final number of subjects who completed the study is reported in Table 1. All subjects were recruited from the claudication clinic at the Nebraska and Western Iowa Veterans Affairs Medical Center (VAMC) in Omaha, Nebraska. The participants answered medical history questions and were evaluated via computed tomographic angiography, physical examination, observational and direct analysis of walking impairments, and hemodynamic analysis [32]. Subjects were enrolled in the study if they could provide written informed consent, had a positive history of claudication, had an ankle-brachial index level below 0.9, and maintained stable blood pressure, lipid, and diabetes regimens, as well as risk factor control for six weeks. A vascular surgeon diagnosed exercise-limited claudication via history and direct observation while monitoring a walking test. The exclusion criteria for this study were: Pain during rest or tissue loss because of PAD, acute ischemia in the legs as a secondary event to acute trauma or thromboembolic disease, and limited walking capacity due to conditions other than claudication, including leg (joint/musculoskeletal, neurologic) and systemic (heart, lung disease) pathology. According to the guidelines outlined in the Declaration of Helsinki, this study was approved by the Ethics Committees of each respective IRB (Protocols #0485–16 and #1576199).

### Data Collection:

All data were collected in three sessions, each three months apart, at the University of Nebraska at Omaha gait laboratory (baseline, three months, six months). The study coordinator performed a simple randomization to assign the patients with PAD to either the control or intervention groups (Figure 1). Following a cross-over study design, the control-first group underwent the standard of care, which did not include an AFO intervention during the initial three months. Subsequently, the subjects assigned to the control-first group completed the AFO intervention the following three months. Conversely, the intervention-first group participated in the three-month AFO intervention, then, after the three-month assessment, they followed a standard of care that did not include wearing AFOs. The cross-over design was the strength of the study as it controls the natural changes that could occur over time with no intervention. In addition, the cross-over study design allowed us to reduce intersubject variability and covariates in this study [33]. During the three-month intervention period, the patients with PAD were instructed to wear the AFOs at all times except when sleeping or showering and follow the typical instructions provided by clinicians to walk at least 30 min per day on most of the days/week. Demographic information for both groups was measured at baseline, three months, and six months.

During all three assessment sessions, subjects wore form-fitting suits and walked with (AFO) and without (non-AFO) bilateral AFOs regardless of their group assignment (Figure 2A,B). Based on the collaborating Orthotist’s recommendation, patients wore either Trulife Matrix or Ottobock WalkOn Reaction AFOs [29,34–37]. AFOs were properly fitted and adjusted at the baseline visit and during the data collection to minimize the risk of sores and discomfort from the device [34–36]. Eight piezoelectric force platforms (Advanced Mechanical Technology, Inc.; Watertown, MA, USA) embedded in a ten-meter walkway were used to measure three-dimensional GRF data at a rate of 1000 Hz as subjects walked across the walkway at a self-selected pace (Figure 2B). During the walking trials, each patient was tested before they began experiencing claudication pain (pain-free), with a mandatory one-minute break between each walking trial to ensure all trials were performed pain-free. Each trial continued until 5 successful heel strikes, and toe-off was recorded (Figure 2B,C).

### Data analysis:

Based on the severity of claudication symptoms, the most affected limb was chosen for analysis [8]. Peak GRFs were analyzed and normalized to body weight in the anterior-posterior, medial-lateral, and vertical (F_z_) directions [4]. In the anterior-posterior direction, peak braking GRF refers to the negative peak after the heel strike [4], and peak propulsion GRF refers to the positive peak before toe-off [38]. In the anterior-posterior direction, the braking impulse refers to the time integral of GRF from touchdown to mid-stance of the gait cycle. In contrast, propulsion impulse refers to the time integral of GRF from the midstance to the toe-off phase of the gait cycle [4,38]. In the medial-lateral direction, the minimum peak is the lateral GRF, and the maximum is the medial GRF. In the vertical direction, we identified the first peak (F_z_1), the second peak (F_z_2), and the local minimum between those two peaks (F_z_min) [4]. The data were processed in Cortex Software version 8.1.4.2039 (Motion Analysis Corp, Santa Rosa, CA, USA; 1000 Hz) and Visual 3D software version v2024.07.2 (C-Motion, Inc., Germantown, MD, USA). Also, a custom program in MATLAB R2017b (The MathWorks Inc., Natick, MA, USA) was used to analyze the raw force plate data. A second-order Butterworth filter with a 12 Hz cut-off frequency was applied to the raw GRF data to smooth the force plate data [39]. All the GRFs were also normalized based on body mass.

### Statistical analysis:

Wilcoxon Rank Sum tests were used to determine differences in baseline data between the control-first and intervention-first groups based on the medians and interquartile ranges (IQRs) of the sample. Two separate, 3-factor linear mixed models were used for GRF statistical analysis: 1. Pre- vs. post-intervention: We examined the impact of using AFOs after the AFO intervention arms in both the control-first (three-month versus six-month assessments) and intervention-first groups (baseline versus three-month assessments; Figure 3A). 2. Intervention vs. no intervention visits: We examined the effect of undergoing an intervention compared to having no intervention (control) by comparing the GRF data at baseline and three months for both the control (no AFO intervention) and intervention (three months of wearing AFOs) groups (Figure 3B).

For GRF data in each direction (i.e., anterior-posterior, medial-lateral, and vertical), separate repeated measures models, with repeated subjects nested within group and unstructured by compound symmetry Kronecker product covariances for time and condition, respectively, were used for each outcome of interest. We included the following factors for the pre- vs. post-intervention comparisons: **Condition:** Walking with (AFO) vs. without AFOs (non-AFO), **Group:** Immediate vs. Delayed intervention, and **Time:** Pre-intervention vs. post-intervention, and the condition by time interaction. For the intervention vs. no intervention comparisons, the models included: **Condition:** Walking with (AFO) vs. without AFOs (non-AFO), **Group:** Control vs. Intervention, and **Time:** Baseline vs. Three months, and all possible interactions. Interactions were removed using backward selection, starting with the highest-ordered interaction and moving on to the highest *p*-value interactions until only interactions with *p*-values less than 0.05 remained; main effects were always retained. Post hoc pairwise comparisons of significant interactions were performed after the Bonferroni adjustment. SAS software version 9.4 (SAS Institute Inc., Cary, NC, USA) was used to conduct the statistical analysis.

## 3. Results

The outcomes of the pre- and post-intervention comparison and intervention vs. no-intervention comparison are provided below. The GRF outcomes were analyzed in three directions in each comparison model: anterior-posterior, medial-lateral, and vertical directions. Additional details regarding the GRFs in the pre- to post-intervention comparison model and the intervention vs. no-intervention models are provided in the Supplementary Materials. Supplementary Materials Figure S1 details the effect of a three-month intervention on GRF across the gait cycle during walking with and without AFOs. Supplementary Materials Figure S2 provides the details for the effect of a three-month AFO intervention vs. no intervention on GRFs, when walking with and without AFOs during the gait cycle.

### 1. Pre- vs. post-intervention comparison:

Fourteen patients with PAD in the control-first group and five patients with PAD in the intervention-first group completed both pre- and post-visits (Figure 1, Table 1). In this comparison, the control-first group was assessed at three and six-month visits, and the intervention-first group was assessed at baseline and three-month visits. In the baseline visit, no significant differences between the control- and intervention-first groups were observed (Table 2).

#### 1. A. Anterior-posterior GRF:

There were no significant effects of the intervention (*p* = 0.3), condition (*p* = 0.4), or group (*p* = 0.6) factors on peak braking GRF (Figure 4A). Similarly, the intervention (*p* = 0.5), condition (*p* = 0.4), or group (*p* = 0.4) factors had no significant effects on braking impulse GRF (Figure 4B). However, we observed a significant effect of condition on peak propulsion GRF (*p* < 0.01). The peak propulsion GRF in both groups in the anterior-posterior direction was reduced significantly while walking with AFOs (1.23, SE = 0.05) compared to walking without AFOs (1.37, SE = 0.05; Figure 4C). There were no significant effects for intervention (*p* = 0.9) or group (*p* = 0.6) factors on peak propulsion GRF. There were no significant effects of intervention (*p* = 0.9), condition (*p* = 0.2), or group (*p* = 0.9) factors on propulsion impulse (Figure 4D).

#### 1. B. Medial-lateral GRF:

##### Medial:

Results showed that the peak medial GRF during walking with AFOs was significantly greater (0.65, SE = 0.03) compared to walking without AFOs (0.61, SE = 0.03; *p* = 0.02; Figure 5A). There were no significant effects of intervention (*p* = 0.2) or group factors on medial GRF (*p* = 0.3).

##### Lateral:

The peak lateral GRF during walking with AFOs (*−*0.26, SE = 0.03) was significantly reduced compared to walking without AFOs (*−*0.31, SE = 0.03; *p* = 0.03; Figure 5B). There were no significant effects of intervention (*p* = 0.7) or group (*p* = 0.7) factors on lateral GRF.

#### 1. C. Vertical GRF:

The F_z_1 (10.38, SE = 0.15) was significantly greater during walking with AFOs compared to walking without AFOs (10.16, SD = 0.15; *p* < 0.01; Figure 6A). There were no significant effects of intervention (*p* = 0.5) or group factors (*p* = 0.1) on F_z_1 GRF. There were no significant effects of the intervention (*p* = 0.1), condition (*p* = 0.06), or group (*p* = 0.8) factors on F_z_min (Figure 6B) or on F_z_2 (intervention *p* = 0.9; condition (*p* = 0.06); or group (*p* = 0.7; Figure 6C).

### 2. Intervention vs. no intervention comparison:

Seventeen patients with PAD in the control-first group and five patients with PAD in the intervention-first group completed the intervention vs. no intervention comparison (Table 2). Both groups were assessed at baseline visits and three-month visits. The group sizes are different due to uneven subject withdrawals (Figure 1). There were no significant differences between GRF data for both the control- and intervention-first groups at the baseline visit (Table 3).

#### 2. A. Anterior-posterior GRF:

The results indicated a two-way interaction between time and condition for peak braking GRF (*p* < 0.01). We used the adjusted magnitudes from the statistical model to ensure any differences at baseline were considered. We observed a significant reduction in peak braking GRF while walking with AFOs (*−*1.12, SE = 0.07) compared to walking without AFOs (*−*1.27, SE = 0.07; *p* = 0.01). However, there was no significant difference between the peak braking GRF in the control and the intervention groups at the three-month visit (Figure 7A; *p* = 0.66). Similarly, a two-way interaction between time and condition was observed for braking impulse (*p* < 0.01). During the baseline visit, walking with AFOs (*−*0.21, SE = 0.01) showed significantly decreased braking impulse compared to walking without AFOs (*−*0.24, SE = 0.01; *p* = 0.03). However, the two groups had no significant difference in braking impulse GRFs during the three-month visit (Figure 7B; *p* = 0.14).

There was a three-way interaction between groups (control vs. intervention), time (intervention vs. no intervention), and conditions (AFO vs. non-AFO) for peak propulsion GRF (*p* < 0.01). Subsequently, two separate models were run for each control and intervention group (group*condition, group*time, time*condition) to find the interactions. There were no significant interactions in the control group (*p* = 0.49). The fixed-effects model showed a main effect of condition when walking with AFOs (*p* < 0.01). The results demonstrated that the control group exhibited a significantly smaller peak propulsion GRF during walking with AFOs (1.22, SE = 0.05) compared to walking without AFOs (1.31, SE = 0.05). There was a significant interaction between time and condition in the intervention group (*p* = 0.01). At the baseline visit, the intervention group had a significantly smaller peak propulsion GRF during walking with AFOs (1.17, SE = 0.10) compared to walking without AFOs (1.42, SE = 0.10; *p* = 0.01). However, there was no significant difference between walking with and without AFOs at the three-month visit in the intervention group (*p* = 1.00) (Figure 7C). We also observed a three-way interaction in the propulsion impulse in the anterior-posterior direction between groups, time, and conditions (*p* = 0.02). However, when conducting pairwise comparisons, no significant differences were detected (Control group: Time*condition interaction (*p* = 0.69), Intervention group: Time*condition interaction (Figure 7D; *p* = 0.55).

#### 2. B. Medial-lateral GRF:

##### Medial:

There were no significant effects for intervention (*p* = 0.6), condition (*p* = 0.07), group (*p* = 0.2), or factors on medial peak GRFs (Figure 8A).

##### Lateral:

No significant effects of intervention (*p* = 0.8), condition (*p* = 0.2), or group (*p* = 0.6) factors were observed on lateral GRF (Figure 8B).

#### 2. C. Vertical GRF:

Walking with AFOs had a significant effect on F_z_1 (*p* < 0.01). At both baseline and the three-month visit, we observed a greater F_z_1 when walking with AFOs (9.97, SE = 0.10) compared to walking without AFOs (9.78, SE = 0.10; *p* < 0.05; Figure 9A). There were no significant effects of intervention (*p* = 0.4), condition (*p* = 0.09), or group (*p* = 0.7) factors on F_z_min (Figure 9B). Walking with AFOs (9.97, SE = 0.10) led to a significantly greater F_z_2 (9.97, SE = 0.10) compared to walking without AFOs (*p* < 0.01, 9.78, SE = 0.10; Figure 9C). There were no significant effects of intervention (*p* = 0.6) or group (*p* = 0.9) factors on F_z_2.

No adverse harm or injuries were reported by the participants during the clinical trial.

## 4. Discussion

The results of this study provide an understanding of how GRFs change when patients with PAD walk with AFOs immediately and following a three-month intervention [4,27]. We hypothesized that implementing a three-month AFO intervention would enhance push-off capabilities by improving the propulsion GRF in the anterior-posterior direction during walking. We also expected that walking with AFOs would improve the propulsion force by reducing calf muscle demand in the anterior-posterior GRF direction. Additionally, we anticipated that walking with AFOs would drive medial-lateral and vertical GRF profiles to improve, as indicated by moving GRFs in patients with PAD towards values comparable to older controls without PAD. Overall, our hypotheses were partially supported.

Regarding the anterior-posterior direction, peak propulsion GRF was reduced while walking with AFOs versus non-AFO (pre-to-post comparison model). This finding aligns with previous studies showing that walking with AFOs reduces propulsion forces [40–42]. Previous studies showed that AFOs reduce ankle range of motion and plantar flexor output to generate propulsion forces [40–42]. Patients with PAD have previously been reported to have kinetic gait impairments in the anterior-posterior direction, especially during the push-off phase of the gait cycle, and even before the onset of claudication [4]. Generally, patients with PAD produced smaller peak propulsion GRF compared to individuals without PAD, and they tend to have a smaller propulsion peak even before the onset of claudication [4,43]. These results indicated a significant reduction in propulsion GRF during walking with AFOs in patients with PAD. It has been shown that the type of AFO used in this study, with a large frontal shin plate that distributes the pressure through the anterior leg, can reduce calf muscle activity [44]. Moreover, the AFOs are designed to stabilize and control the ankle joint, which can limit its natural range of motion during walking [45]. Alternatively, the AFO can absorb energy during heel strike and release it during push-off [29–31]. Thus, the AFOs have the potential to assist patients during push-off, potentially allowing patients with PAD to rely less on their muscles to produce propulsive GRF. In the intervention-first group (intervention vs. no intervention model), the propulsion peak GRF significantly increased after a three-month AFO intervention. This suggests that the AFO intervention helped patients with PAD and improved patients’ ability to walk with AFOs in a manner that led to greater propulsion GRF. Overall, we saw that the three-month AFO intervention led to an improved peak propulsion GRF, even though the acute impact of wearing AFOs was to decrease propulsion GRF.

For peak braking GRF, patients with PAD exhibited decreased braking GRF and braking impulse during walking with AFOs compared to non-AFO at baseline (intervention vs. no intervention comparison model). However, following both the control period and intervention period, a significant difference between walking with AFOs and non-AFOs was not observed. This indicates that the patients adapted to walking with AFOs over time. Previous research reported that braking impulse was positively correlated with knee extensor moments [9]. Our previous data analysis revealed that both the control and the intervention-first group had a smaller, though not statistically significant, knee extensor moment at the baseline visit [36]. A three-month AFO intervention caused an increase in knee extensor moment in both control and intervention groups [36]. As a result, the lower braking GRF at baseline visits may result from reduced knee extensor contribution that increased after a three-month intervention with AFOs [36].

In the medial-lateral GRF direction, our results demonstrated that patients with PAD had a lower peak lateral GRF during walking with AFOs compared to non-AFO. In previous studies, patients with PAD exhibited rapid plantar flexion or foot drop after the initial heel strike [46]. Patients with PAD have also shown an association between foot drop and medial-lateral stability reduction, which may cause muscle fatigue. AFOs are assistive devices commonly prescribed to improve ankle-foot stability and reduce foot drop [47–50]. Studies have demonstrated a larger lateral and smaller medial GRF due to ankle-foot instability [51,52]. So, it is possible that the reduced lateral GRF and increased medial GRF while walking with AFOs could lead to increased ankle-foot stability [9,49,52].

In the vertical GRF direction, patients with PAD increased F_z_1 (pre to post and intervention vs. no intervention comparison models) and F_z_2 (intervention vs. no intervention comparison model) during walking with AFOs compared to non-AFO conditions. These findings align with a previous study that walking with AFOs increases F_z_1 and F_z_2 in children with cerebral palsy [53]. Previous work reported that patients with PAD showed a flatter vertical GRF curve, indicating less center of mass fluctuation compared to healthy subjects [4,54]. Vertical fluctuation reduction reflects increased double-limb support time and the inability to extend the leg fully during single-limb support [4]. Extending the previous findings, walking with AFOs seemed to increase vertical fluctuations. These changes may reflect energy storage and return in the AFO and support full leg extension during the single-limb support phase, making the vertical GRF pattern comparable to that of individuals without PAD [36,46,50,51].

### Limitations

This study had some limitations. Potential learning for the subjects from the baseline visit might affect their walking pattern in the three-month and six-month visits. We attempted to mitigate this with the control-crossover design. Our subject populations are also limited by primarily being male subjects due to recruitment from our local Veterans Affairs medical center, which restricts our findings from being generalized to other populations, such as females or non-veterans. The unequal distribution of the control-first and intervention-first groups was due to subject withdrawal. During the study period, the final total number of subjects in the intervention-first group was fewer than in the control-first group, due to a higher number of withdrawals in the intervention-first group. Therefore, the results should be interpreted cautiously. The unequal distribution of participants using Trulife Matrix and Ottobock WalkOn Reaction AFOs was another limitation of this study. The difference between the Matrix Trulife and Ottobock WalkOn Reaction mechanical properties and stiffness might impact the participant’s walking performance [41,55]. However, a previous study suggested that the AFO stiffness tends to impact gait kinematics more than gait kinetics [56]. Both AFO types used in this study have the same clinical intent (carbon, composite, non-articulate, assist push-off, and control ankle motion) [34,36,41,44]. Additionally, AFO stiffness has a minimal effect on GRFs, including F_z_1, F_z_min, F_z_2, and braking GRF [56–58], while it has a greater impact on medial-lateral GRF and propulsion force [57]. Therefore, these results should be interpreted with caution. Patients with PAD in this study walked at self-selected walking speed, and we did not include the walking speed as a covariate in the statistical analysis. Since the primary goal of this manuscript was to assess the long-term effect of AFOs on the GRF of patients with PAD, and not directly compare the effect of AFO stiffnesses or control the walking speed, future studies are necessary to compare the long-term effect of the type of AFOs while controlling the walking speed on the walking performance of patients with PAD.

## 5. Conclusions

This research has increased our understanding of the effect of a three-month AFO intervention on the GRFs of patients with PAD. To the best of our knowledge, this study represents the first comparison between pre- and post-AFO intervention on GRF during walking in patients with PAD. In summary, the critical analysis of our study showed that reduced propulsion force during walking with an AFO compared to without an AFO, potentially allowing participants to rely less on their calf muscles to produce propulsion GRF. Overall, we observed that a three-month AFO intervention increased GRF propulsion, even though the acute impact of wearing AFOs was to decrease GRF propulsion. Moreover, walking with AFOs increased medial GRF and reduced lateral GRF, suggesting ankle support based on the medial-lateral GRFs during walking in patients with PAD. Walking with AFOs also increased vertical GRFs toward the trend previously reported in healthy individuals. AFOs have an overall positive effect on the GRF profiles of patients with PAD. These findings support considering AFOs as a biomechanically assistive device option for patients with PAD to improve gait kinetics. These findings provide several directions for future studies, including examining the effects of various AFO designs, materials, and stiffness levels on gait kinetics in patients with PAD. In addition, future studies could assess the association between muscle activation during walking with AFOs and GRF in patients with PAD to clarify how AFOs affect gait kinetics.

## Supplementary Material

Supplementary Material

The following supporting information can be downloaded at: https://www.mdpi.com/article/10.3390/act15040187/s1, Figure S1 shows the effect of a three-month AFO intervention on GRFs when walking with and without AFOs across the gait cycle. Additionally, Figure S2 shows the effect of a three-month AFO intervention vs. no intervention on GRFs when walking with and without AFOs during a gait cycle.

## Figures and Tables

**Figure 1.**
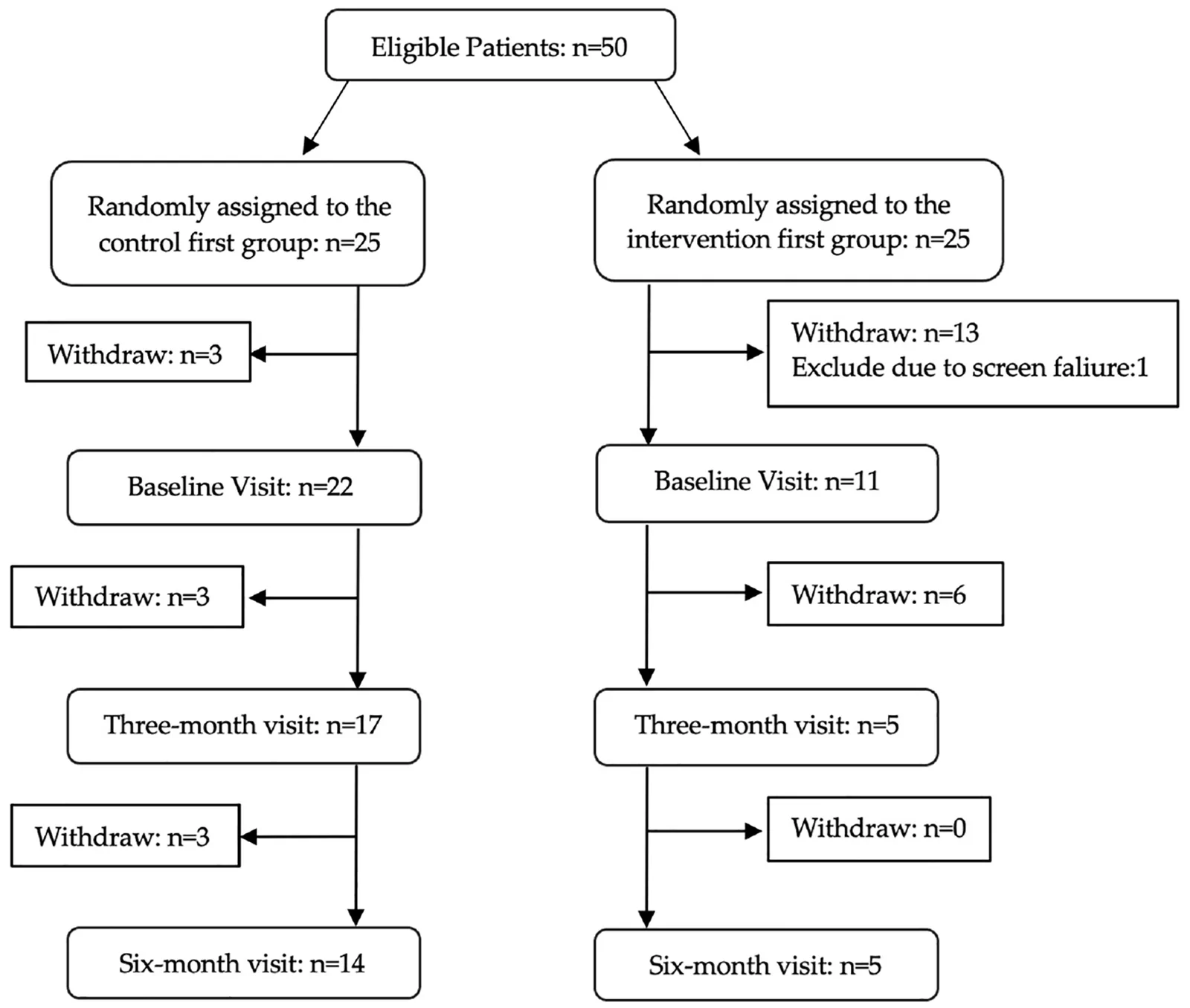
Screening flow diagram.

**Figure 2.**
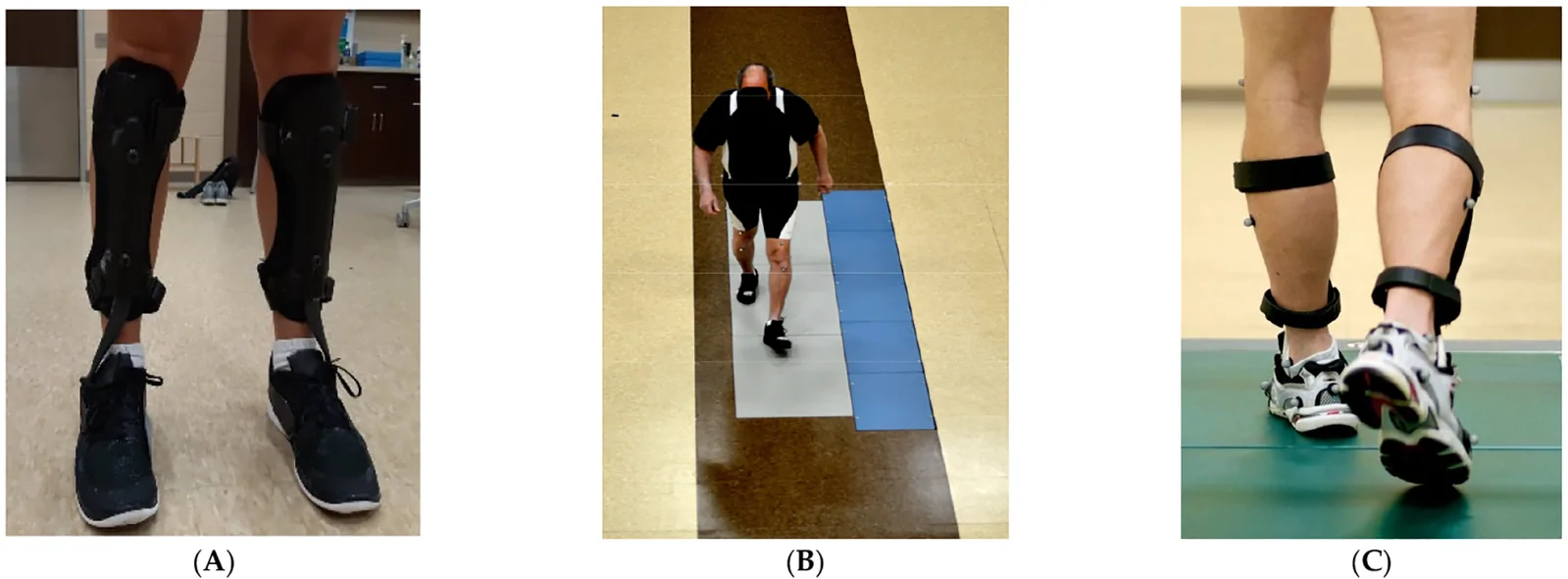
(**A**) Subjects wore ankle foot orthoses (AFOs) on both legs; (**B**) participants walked over ground with and without AFO until five successful heel strike and toe-off events were recorded; (**C**) walking with AFOs on both legs.

**Figure 3.**
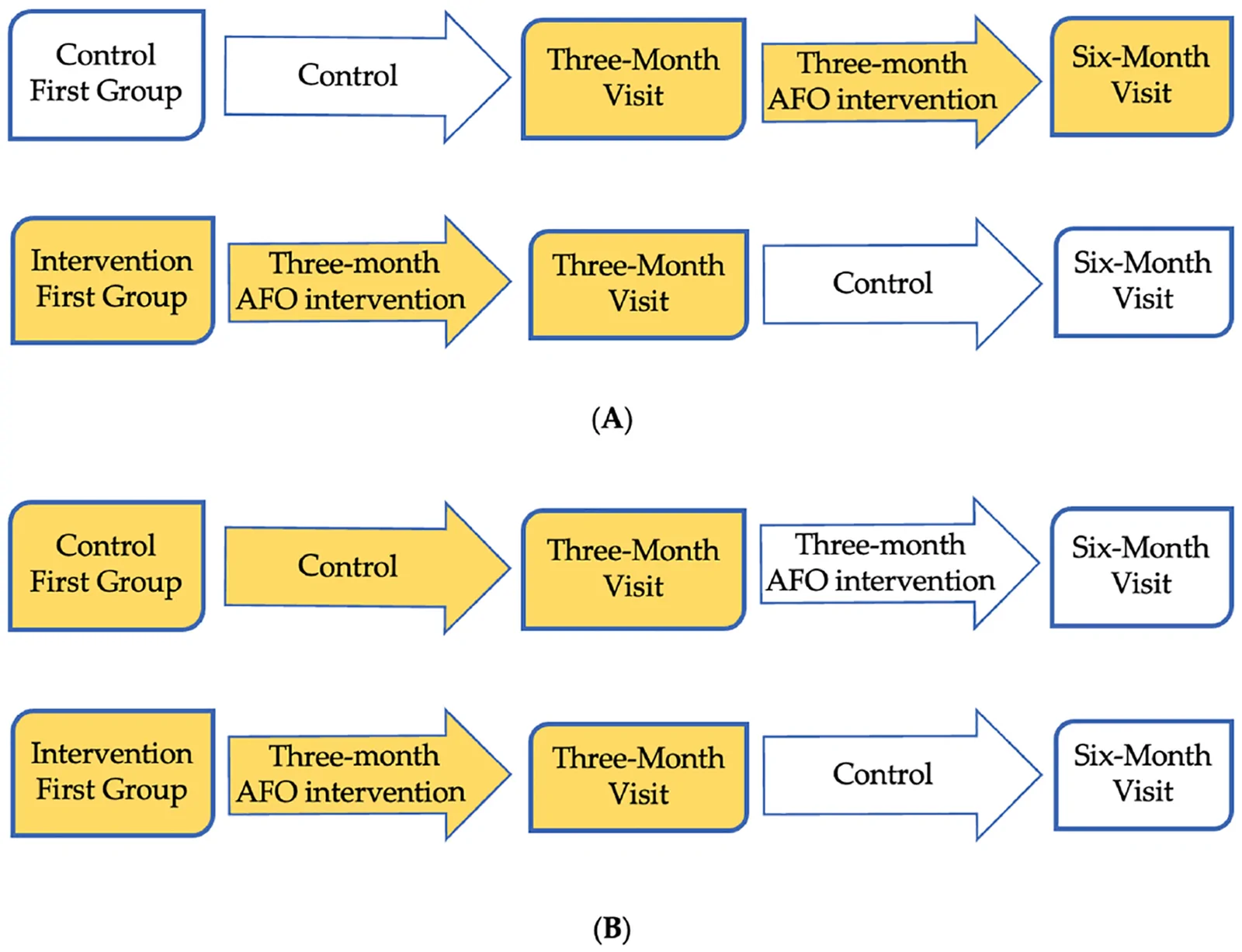
Group interpretations are provided based on the crossover design of the study. Control First Group: No AFO intervention was received during the first three months. Subsequently, patients with PAD received the AFO intervention during the following three-month period. Intervention First Group: Subjects received the intervention during the first three months and did not undergo any AFO intervention during the subsequent three-month period. (**A**) Pre-Post Intervention Comparison: The yellow outlines compare the dependent variables of the control first group and the intervention first group before and after the intervention. (**B**) Intervention vs. No Intervention Comparison: The yellow outlines compare the baseline and three-month AFO intervention between the control-first and intervention-first groups. Both models included condition, time, and group factors.

**Figure 4.**
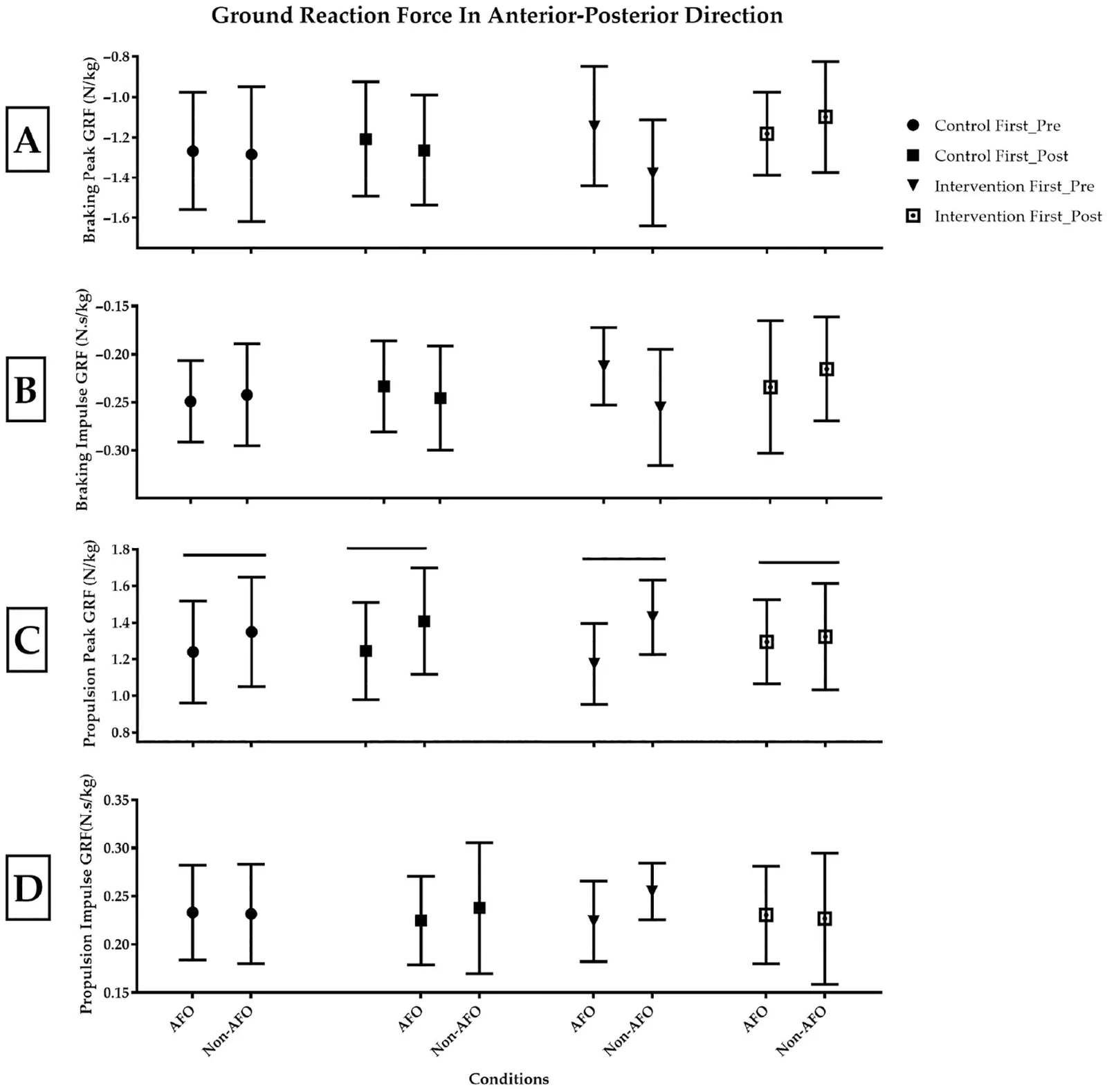
Pre to post-intervention comparison: These figures represent the mean and standard deviation of the peak ground reaction forces (GRFs) in the anterior-posterior direction. The solid horizontal lines represent the significant differences between conditions: (**A**) There was no significant effect of intervention, condition, or group factors on braking peak GRF; (**B**) There was no significant effect of intervention, condition, or group factors on braking impulse ground reaction force (GRF); (**C**) The results showed a significant effect of walking with AFOs on peak propulsion GRF; (**D**) There was no significant effect of intervention, condition, and group factors on propulsion impulse GRF. AFO: Bilateral ankle foot orthosis condition. Non-AFO: Walking without ankle foot orthoses.

**Figure 5.**
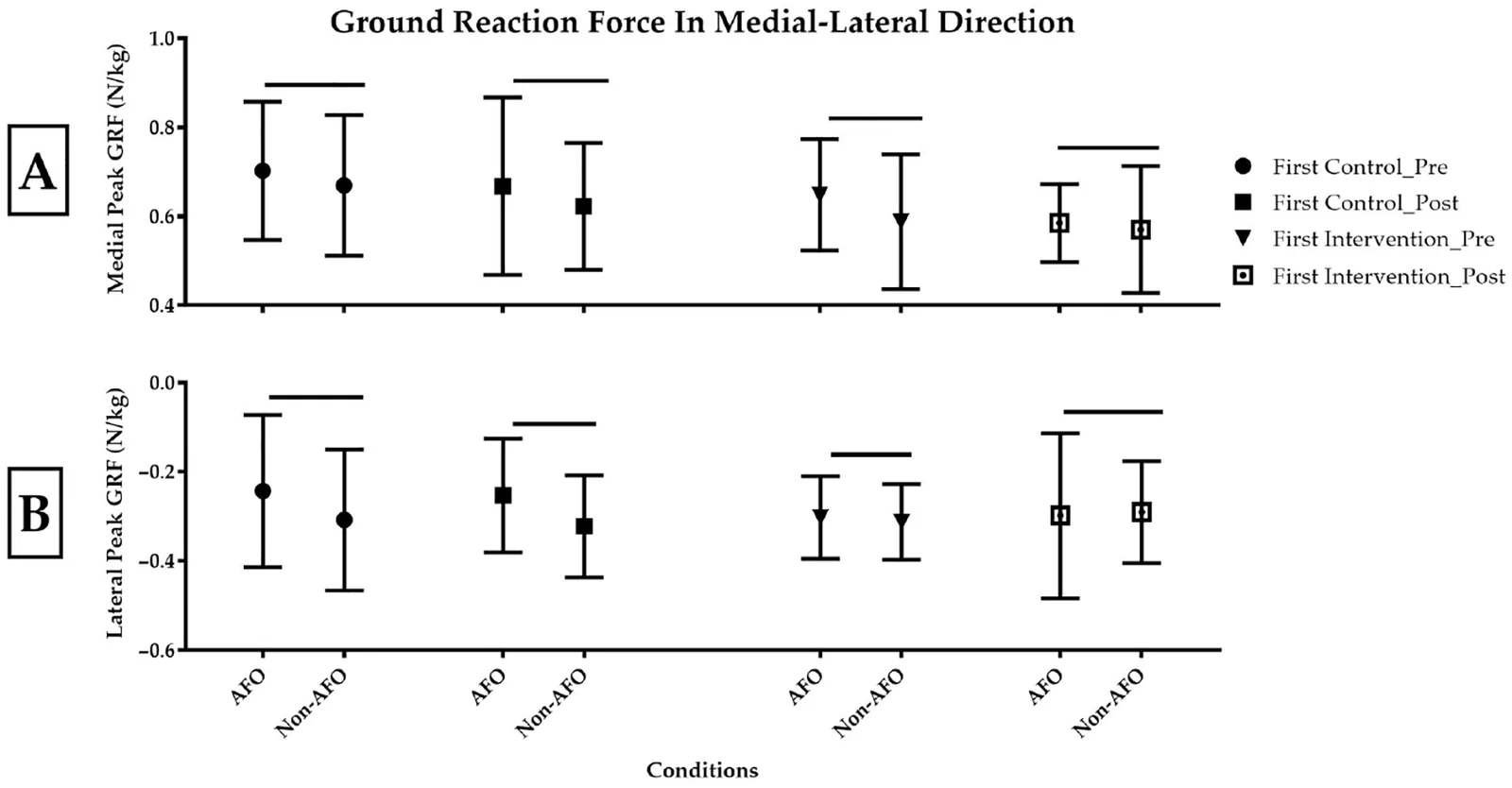
Pre to post-intervention comparison: These figures represent the mean and standard deviation of the peak ground reaction forces (GRFs) in the medial-lateral direction. The solid horizontal lines represent the significant differences between conditions: (**A**) There was a significant effect of walking with AFOs on medial peak GRF in both control first and intervention first groups before and after the intervention; (**B**) There was a significant effect of walking with AFOs on peak lateral GRF between both control first and intervention first groups before and after the intervention. AFO: Bilateral ankle foot orthosis condition. Non-AFO: Walking without ankle foot orthoses.

**Figure 6.**
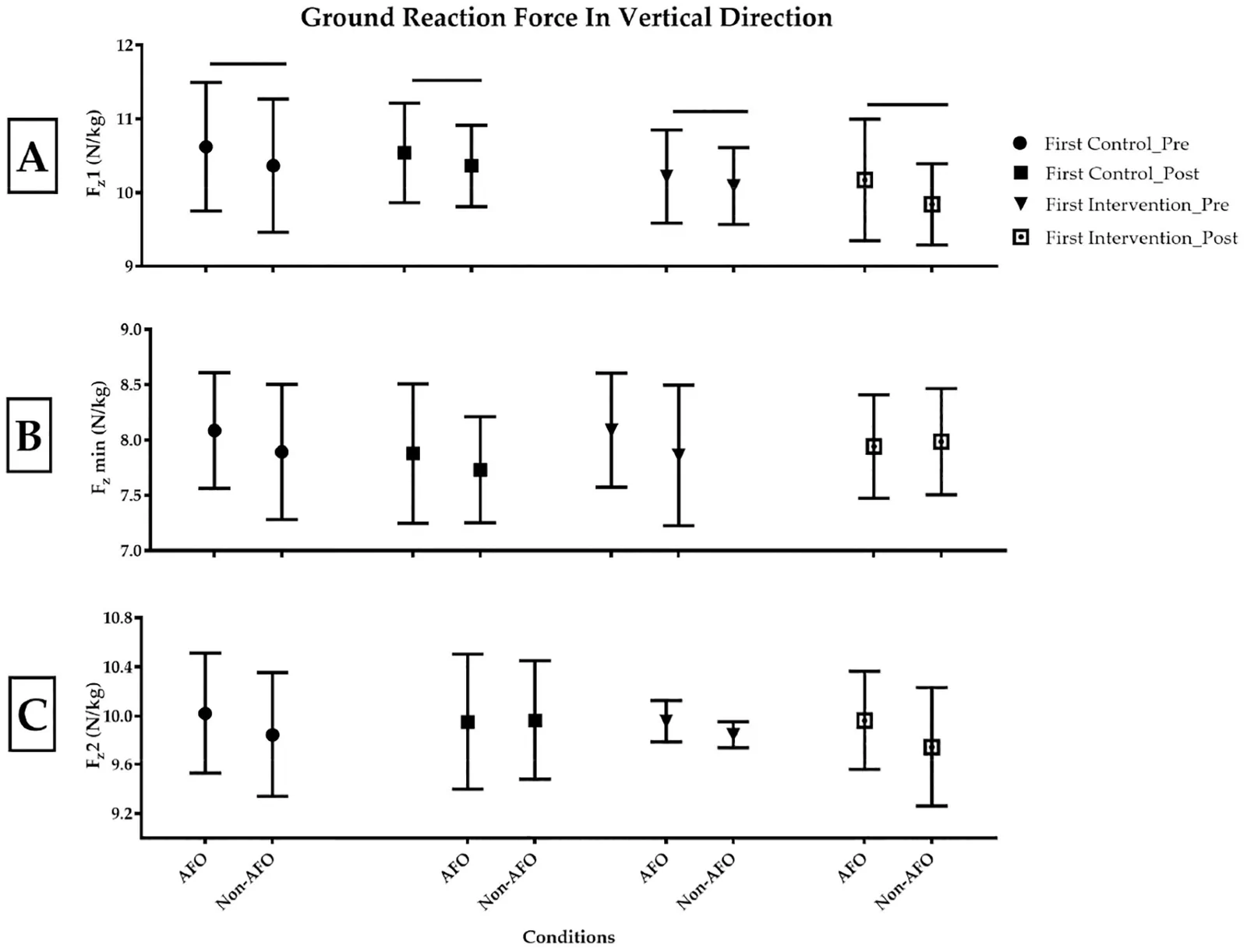
Pre to post-intervention comparison: These figures represent the mean and standard deviation of the peak ground reaction forces (GRFs) in the vertical direction. The solid horizontal lines represent the significant differences between conditions: (**A**) There was a significant effect of walking with AFOs on the first maximum peak vertical ground reaction force (F_z_1); (**B**) There was no significant effect of intervention, condition, and group factors on the local minimum between the first two peaks (F_z_min); (**C**) There was no significant effect of intervention, condition, and group factors on the second maximum peak vertical GRF (F_z_2). AFO: Bilateral ankle foot orthosis condition. Non-AFO: Walking without ankle foot orthoses.

**Figure 7.**
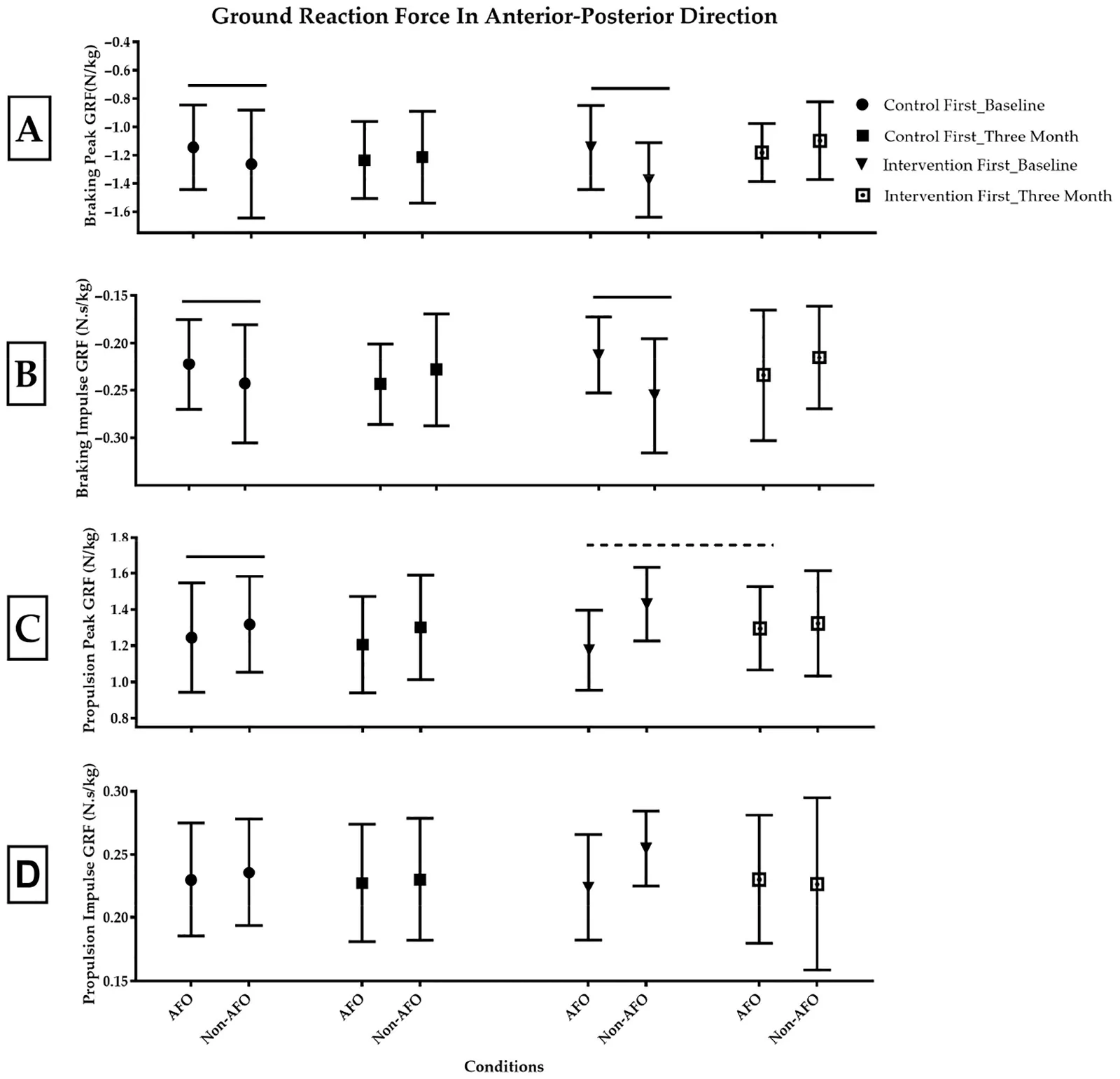
Intervention vs. no intervention comparison: These graphs compare the mean and standard deviation of the anterior-posterior ground reaction forces (GRFs) at the baseline and three-month AO intervention visits of the control first and the intervention first groups. The solid horizontal lines represent the significant differences between conditions. The dashed line shows the significant difference for the intervention factor: (**A**) There was a significant effect of walking without an AFO on braking peak GRF; (**B**) There was a significant effect of walking without AFOs on braking impulse GRF; (**C**) There was a significant effect of walking with AFOs on propulsion peak GRF at the baseline visit of the control first group; (**D**) There was no significant effect of intervention, condition, or group factors on propulsion impulse GRF. AFO: Bilateral ankle foot orthosis condition. Non-AFO: Walking without ankle foot orthoses.

**Figure 8.**
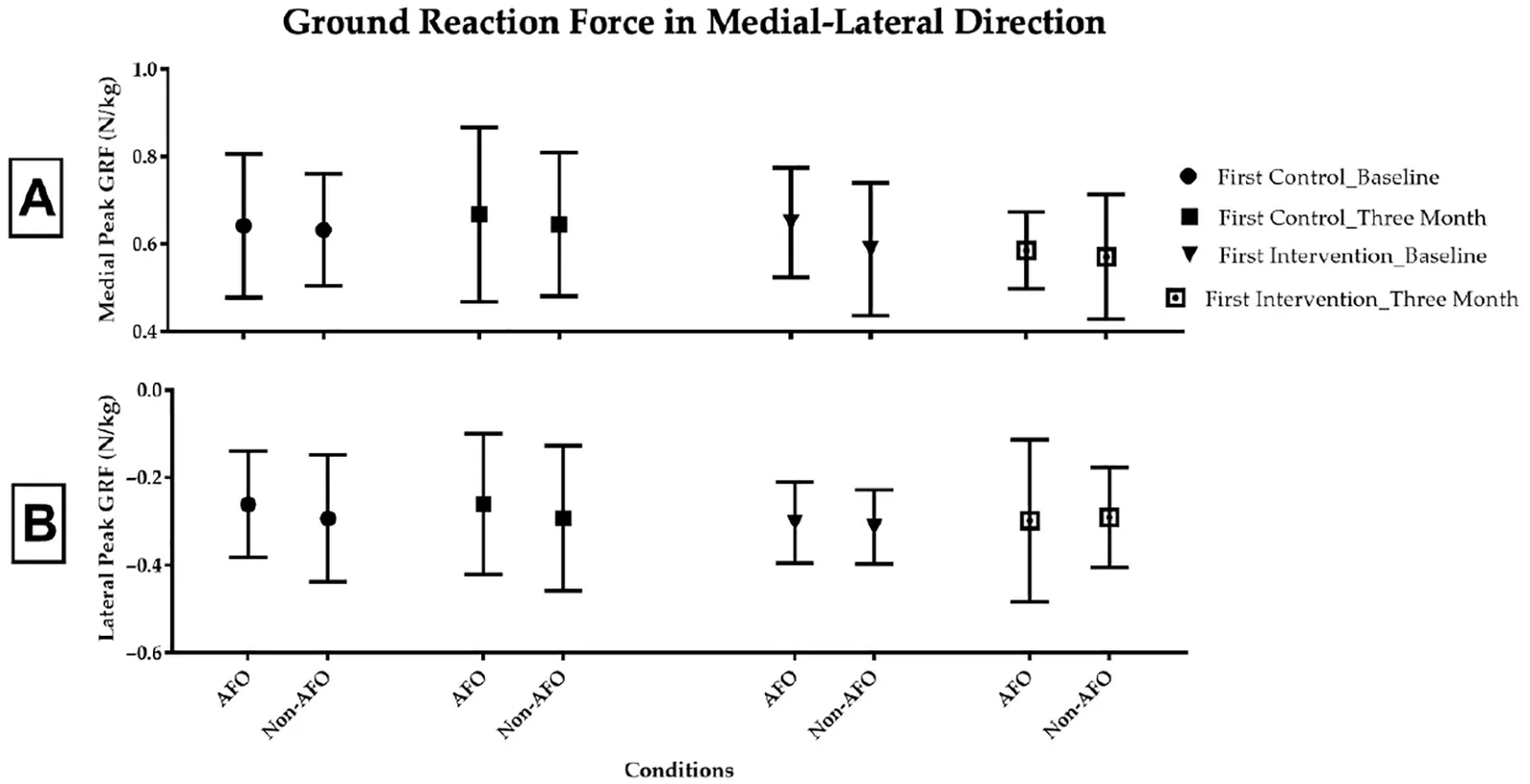
Intervention vs. no intervention comparison: These graphs compare the mean and standard deviation of the medial-lateral ground reaction forces (GRFs) at the baseline and three-month AFO intervention visits of the control first and the intervention first groups. The solid horizontal lines represent the significant differences between conditions: (**A**) There was no significant effect of intervention, condition, or group factors on medial GRF; (**B**) There was no significant effect of intervention, condition, or group factors on lateral GRF. AFO: Bilateral ankle foot orthosis condition. Non-AFO: Walking without ankle foot orthoses.

**Figure 9.**
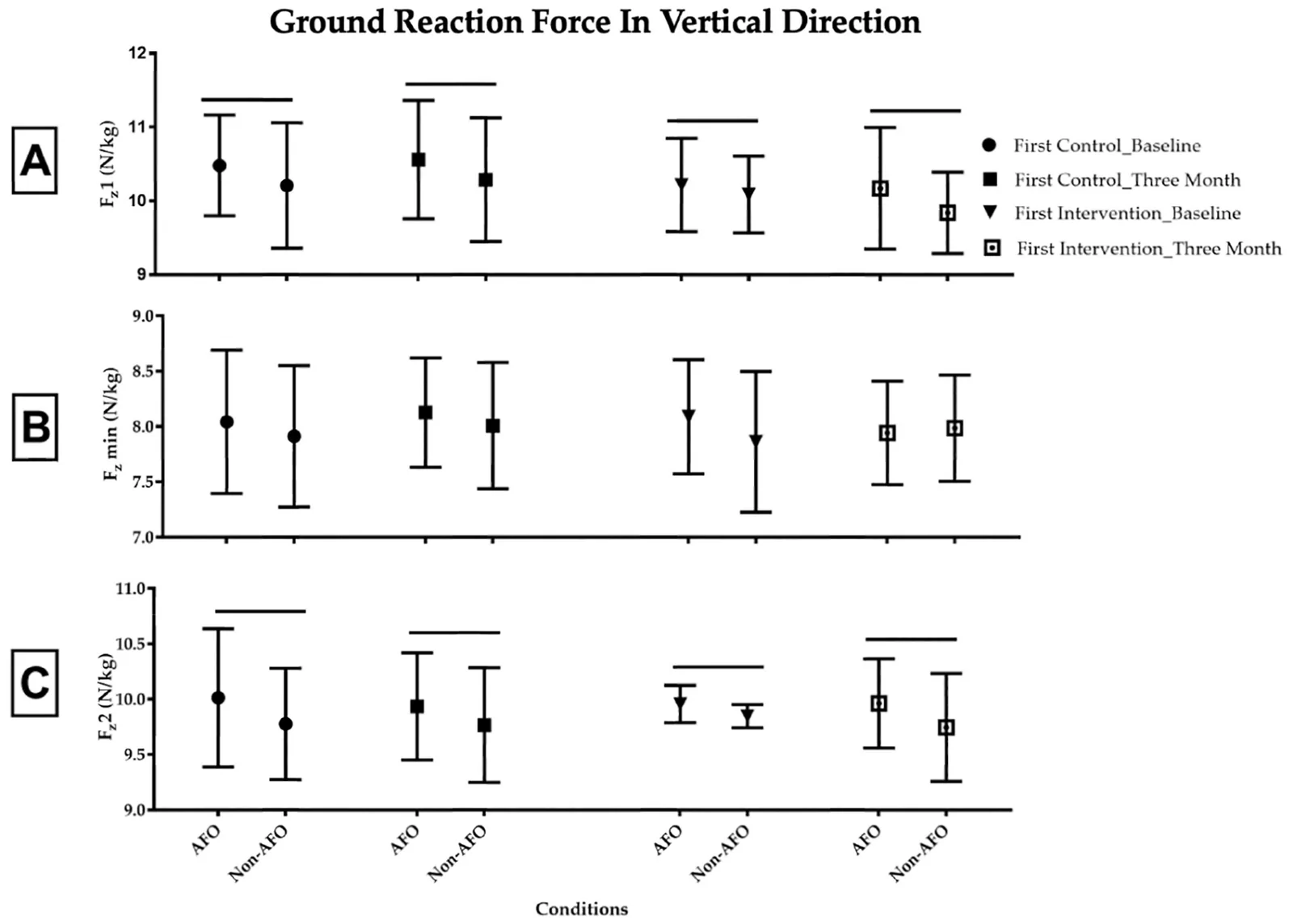
Intervention vs. no intervention comparison: These graphs compare the mean and standard deviation of the vertical ground reaction forces (GRFs) at the baseline and three-month AFO intervention visits of the control first and the intervention first groups. The solid horizontal lines represent the significant differences between conditions: (**A**) There was a significant effect of walking with AFOs in both the control first and intervention first groups on the first peak vertical GRF (F_z_1); (**B**) There was no significant effect on intervention, condition, or group factors on the local minimum between the first two peaks of vertical GRF; (**C**) There was a significant effect of walking with AFOs on the second peak vertical GRF(F_z_2) in both control first and intervention first groups. AFO: Bilateral ankle foot orthosis condition. Non-AFO: Walking without ankle foot orthoses.

**Table 1.** Demographic characteristics of the pre-post intervention and intervention vs. no intervention comparison: In the pre-post intervention comparison model, we compared the three-month and six-month visits for the control-first group and the baseline and three-month visits from the intervention-first group. For the intervention vs. no intervention comparison model, both groups were assessed at baseline visits and three-month visits. The group sizes are different due to subject withdrawals. In the control-first group, three subjects withdrew between three- and six-month visits. The Wilcoxon rank-sum test confirmed that there was no significant difference in demographic characteristics between the control-first and intervention-first groups at baseline assessment in both the pre-post intervention and intervention vs. no-intervention comparison models (*p* > 0.05). In the pre-to-post statistical model, the baseline assessment for the control-first group was at the three-month visit, while patients in the intervention-first group were assessed at baseline. In the intervention vs. no-intervention statistical model, the baseline assessment for both intervention-first and control-first groups was at the baseline visit.

Pre–Post Intervention			
	Control-First Group (n = 14)	Intervention-First Group (n = 5)	*p*-value
**Sex (number)**	Male (14)/Female (0)	Male (5)/Female (0)	
**High blood pressure (number)**	10/14	5/5	
**Smoking (number)**	4/14	4/5	
**Diabetes (number)**	6/14	1/5	
**Trulife Matrix AFOs (number)**	10/14	3/5	
**Ottobock WalkOn Reaction AFOs (number)**	4/14	2/5	
**Age (years) Median (IQR)**	71.5 (70.0, 77.0)	70.0 (68.0, 72.0)	0.40
**Body mass index (kg/m^2^) Median (IQR)**	29.7 (26.4, 32.2)	28.6 (22.1, 31.8)	0.43
**Height (cm) Median (IQR)**	175.3 (171.5, 180.3)	177.2 (167.6, 177.8)	0.58
**ABI (ratio) Median (IQR)**	0.7 (0.5, 0.9)	0.6 (0.5, 0.7)	0.40
Intervention vs. No Intervention			
	Control-first Group (n = 17)	Intervention-first Group (n = 5)	*p*-value
**Sex (number)**	Male (17)/Female (0)	Male (5)/Female (0)	
**High blood pressure (number)**	13/17	5/5	
**Smoking (number)**	5/17	4/5	
**Diabetes (number)**	8/17	1/5	
**Trulife Matrix AFOs (number)**	12/17	3/5	
**Ottobock WalkOn Reaction AFOs (number)**	5/17	2/5	
**Age (years) Median (IQR)**	72.0 (70.0, 79.0)	70.0 (68.0, 72.0)	0.22
**Body mass index (kg/m^2^) Median (IQR)**	30.0 (25.7, 32.8)	28.6 (22.1, 31.8)	0.39
**Height (cm) Median (IQR)**	172.7 (171.5, 180.3)	177.2 (167.6, 177.8)	0.66
**ABI (ratio) Median (IQR)**	0.7 (0.5, 0.8)	0.6 (0.5, 0.7)	0.42

ABI: Ankle brachial index.

**Table 2.** The results confirmed no significant differences in ground reaction force (GRF) in all three directions (Anterior-posterior, medial-lateral, and vertical) between the control and intervention groups at baseline in the pre- to post-intervention comparison model (*p* > 0.05).

Pre to Post Intervention Statistical Model							
		Control-First Group		Intervention-First Group		*p*-Value	
		AFO	Non-AFO	AFO	Non-AFO	Walking with AFOs (*p*-values): Control first vs. intervention first	Walking without AFO (*p*-values): Control first vs. intervention first
**Anterior-Posterior**	Brake (N/kg) Median (IQR)	−1.3 (−1.5, 1.06)	−1.2 (−1.5, −0.9)	−0.9 (−1.3, −0.94)	−1.4 (−1.5, −1.2)	0.5	0.4
	Brake Impulse (N·s/kg) Median (IQR)	−20.2 (−26.3, −17.3)	−18.2 (−29.04, −17.8)	−16.8 (−23.4, −14.6)	−26.7 (−28.4, −14.5)	0.2	0.7
	Propulsion (N/kg) Medial Median (IQR)	1.2 (1.09, 1.4)	1.3 (1.1, 1.5)	1.2 (1.04, 1.3)	1.4 (1.4, 1.5)	0.7	0.5
	Propulsion Impulse (N·s/kg) Median (IQR)	20.02 (15.9, 24.1)	20.01 (26.7, 23.9)	22.1 (12.4, 23.2)	23.27 (15.0, 24.1)	0.7	0.6
**Medial-Lateral**	Medial (N/kg) Median (IQR)	0.6 (0.5, 0.8)	0.6 (0.5, 0.7)	0.6 (0.5, 0.7)	0.5 (0.4, 0.7)	0.3	0.3
	Lateral (N/kg) Median (IQR)	−0.2 (−0.3, −0,1)	−0.3 (−0.4, −0.1)	0.09 (−0.3, −0,2)	0.3 (−0.3, −0.2)	0.3	0.8
**Vertical**	F_z_1 (N/kg) Median (IQR)	10.3 (10.1, 10.8)	10.1 (0.8, 10.4)	10.1 (9.9, 10.3)	10.2 (97, 10.4)	0.3	0.8
	F_z_min (N/kg) Median (IQR)	8.1 (7.7, 8.4)	8.09 (7.4, 8.4)	8.2 (7.7, 8.3)	8.2 (7.1, 8.2)	0.9	0.8
	F_z_2 (N/kg) Median (IQR)	10 (9.6, 10.2)	9.7 (9.4, 10.07)	10.03 (9.8, 10.06)	9.8 (9.7, 9.9)	0.9	0.8

AFO: Ankle foot orthosis. Non-AFO: Non-Ankle foot orthosis. F_z_1: The first vertical ground reaction force peak. F_z_2: The second vertical ground reaction force peak. F_z_min: Local minimum between the first and second vertical ground reaction force peaks.

**Table 3.** The results confirmed no significant differences between the control and intervention groups in ground reaction force (GRF) in all three directions (Anterior-posterior, medial-lateral, and vertical) at the baseline visit in the intervention vs. no intervention comparison model (*p* > 0.05).

Intervention vs. No Intervention Statistical Model							
		Control-First Group		Intervention-First Group		*p*-Value	
		AFO	Non-AFO	AFO	Non-AFO	Walking with AFOs (*p*-values): Control first vs. intervention first	Walking without (*p*-values): Control first vs. intervention first
**Anterior-Posterior**	Brake (N/kg) Median (IQR)	−1.0.9 (−1.2, −0.9)	−1.2 (−1.3, −1.03)	−0.9 (−1.3, −0.9)	−1.4 (−1.5, −1.2)	0.7	0.3
	Brake Impulse (N·s/kg) Median (IQR)	−20.2 (−23.06, −30.7)	−22.26 (−27.3, −17.9)	−18.2 (−23.4, −14.6)	−21.9 (−28.4, −14.5)	0.4	0.8
	Propulsion (N/kg) Median (IQR)	1.2 (1.08, 1.3)	1.3 (1.1, 1.4)	1.4 (1.04, 1.3)	1.2 (1.4, 1.5)	1.0	0.3
	Propulsion Impulse (N·s/kg) Median (IQR)	20.7 (15.9, 24.1)	20.8 (16.7, 23.9)	19.4 (12.4, 23.2)	22.02 (15.0, 24.1)	0.7	0.6
**Medial-Lateral**	Medial (N/kg) Median (IQR)	0.6 (0.5, 0.7)	0.5 (0.5, 0.6)	0.6 (0.5, 0.7)	0.5 (0.4, 0.7)	1.0	0.4
	Lateral (N/kg) Median (IQR)	−0.2 (−0.3, −0.2)	−0.3 (−0.3, −0.2)	−0.3 (−0.3, −0.2)	−0.3 (−0.3, −0.2)	0.5	0.8
**Vertical**	F_z_1 (N/kg) Median (IQR)	10.2 (10.1, 10.5)	10.1 (9.6, 10.3)	10.1 (9.9, 10.3)	10.2 (9.7, 10.4)	0.5	0.6
	F_z_min (N/kg) Median (IQR)	8.1 (8.04, 8.4)	8.04 (7.7, 8.2)	8.02 (8.7, 8.3)	8.2 (7.1, 7.1)	0.9	0.9
	F_z_2 (N/kg) Median (IQR)	9.8 (9.6, 10.1)	9.6 (9.4, 10.05)	10.03 (9.8, 10.06)	9.8 (9.7, 9.9)	0.9	0.5

AFO: Ankle foot orthosis. Non-AFO: Non-Ankle foot orthosis. F_z_1: The first vertical ground reaction force peak. F_z_2: The second vertical ground reaction force peak. F_z_min: Local minimum between the first and second vertical ground reaction force peaks.

## Data Availability

The data presented in this study are available on request from the corresponding author due to privacy and ethical restriction.

## Abbreviations

The following abbreviations are used in this manuscript:

PAD: Peripheral artery disease
GRF: Ground reaction force
AP: Anterior-posterior
ML: Medial lateral
AFO: ankle foot orthoses
