# Supplementary material for: Ankle Foot Orthosis Intervention Improves the Ground Reaction Forces During Walking in Patients with Peripheral Artery Disease (Randomized Clinical Trial)
Source: Actuators. Author manuscript; Available in PMC 2026 Nov 7. (PMC13742116; doi:10.3390/act15040187)
Supplement: Supplementary file 1 [file NIHMS2219299-supplement-Supplementary_Material.zip › S1.pdf]

Supplementary material S1

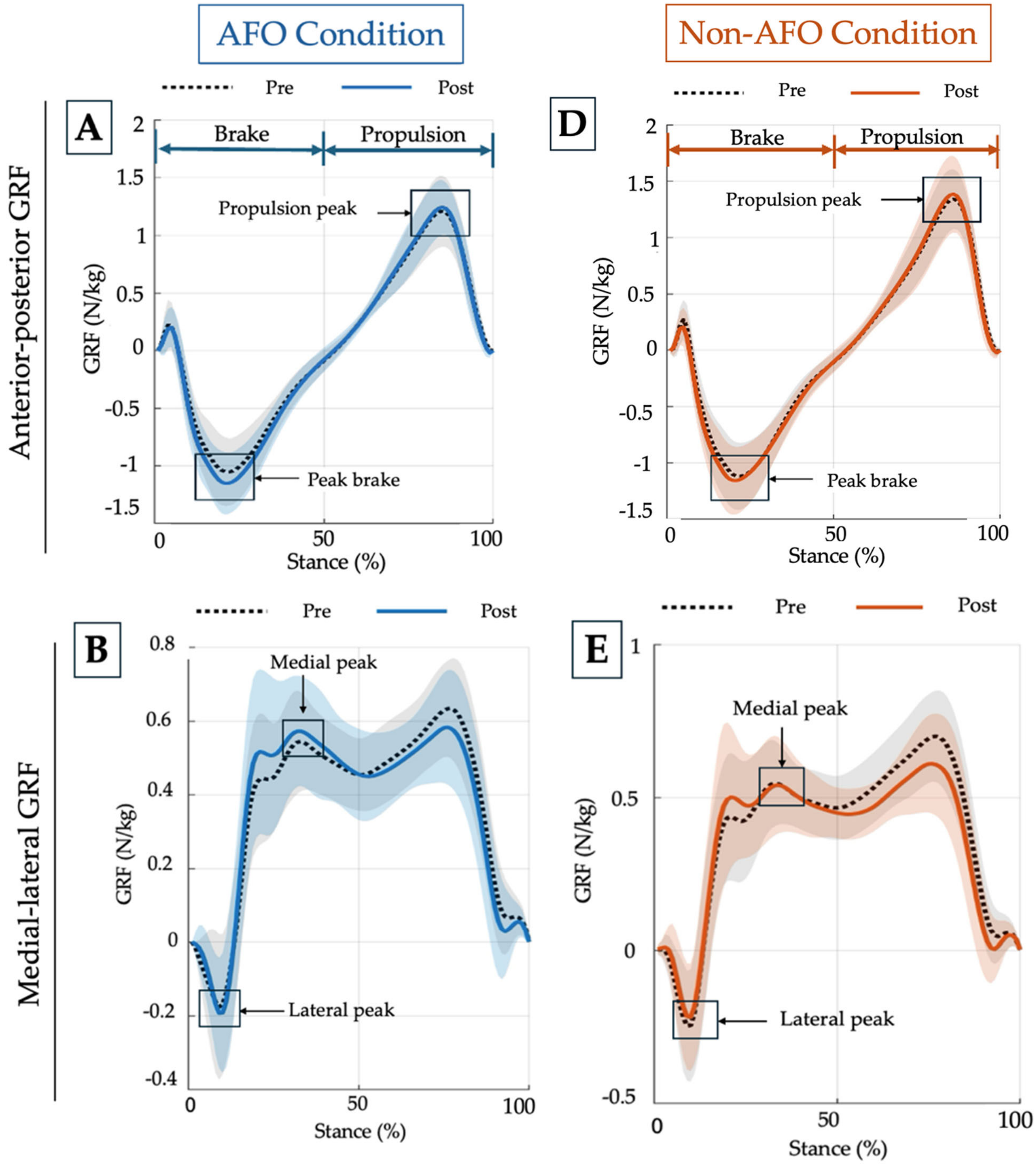

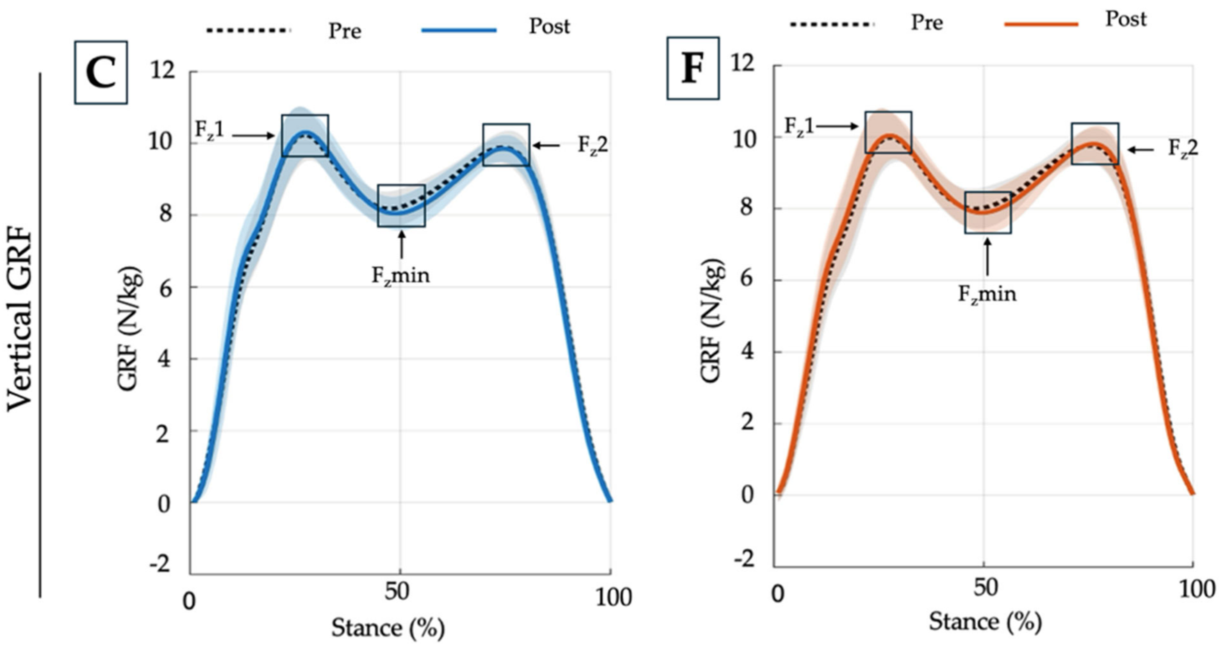

Figure S1. The first control and first intervention groups are combined in this figure. This figure shows the effect of a three-month AFO intervention on GRFs during walking with and without AFOs across the gait cycle. **A)** Anterior-posterior GRF when patients walked with AFOs. **B)** Medial-lateral GRF when patients walked with AFOs. **C)** Vertical GRF when patients walked with AFOs. **D)** Anterior-posterior GRF when patients walked without AFOs. **E)** Medial-lateral GRF when patients walked without AFOs. **F)** Vertical GRF when patients walked without AFOs. **Non-AFO:** Walking without ankle foot orthosis. **GRF:** Ground reaction force. **F_z1:** First maximum peak vertical GRF. **F_z2:** Second maximum peak vertical GRF. **$F_z$min:** Local minimum between peak vertical GRF.
